# Development, implementation and evaluation of a seven-day clinical pharmacy service in a tertiary referral teaching hospital during surge-2 of the COVID-19 pandemic

**DOI:** 10.1007/s11096-022-01475-8

**Published:** 2022-11-11

**Authors:** C. Cheng, A. Walsh, S. Jones, S. Matthews, D. Weerasooriya, R. J. Fernandes, C. A. McKenzie

**Affiliations:** 1 Pharmacy Department, Kings College Hospital, London, SE5 9RS UK; 2 Institute of Pharmaceutical Sciences and Institute of Psychiatry, Psychology, Neurosciences Kings College London, London, SE1 9RT UK; 3 Pharmacy Department, Medway NHS Foundation Trust, Gillingham, ME7 5NY UK; 4 Pharmacy and Critical Care, University Hospital Southampton, Tremona Road, Southampton, S016 6YD UK

**Keywords:** Hospitals, Hospital pharmacy service, Implementation science, Medication reconciliation, Pandemics, Pharmacists

## Abstract

**Background:**

Seven-day clinical pharmacy services in the acute sector of the National Health Service are limited. There is a paucity of evidential patient benefit. This limits investment and infrastructure, despite United Kingdom wide calls.

**Aim:**

To optimise medicines seven-days a week during surge-2 of the COVID-19 pandemic through implementation of a seven-day clinical pharmacy service. This paper describes service development, evaluation and sustainability.

**Setting:**

A tertiary-referral teaching hospital, London, United Kingdom.

**Development:**

The seven-day clinical pharmacy service was developed to critical care, acute and general medical patients. Clinical leads developed the service specification and defined priorities, targeting complex patients and transfer of care. Contributing staff were briefed and training materials developed.

**Implementation:**

The service was implemented in January 2021 for 11 weeks. Multidisciplinary team communication brought challenges; strategies were employed to overcome these.

**Evaluation:**

A prospective observational study was conducted in intervention wards over two weekends in February 2021. 1584 beds were occupied and 602 patients included. 346 interventions were reported and rated; 85.6% had high or moderate impact; 56.7% were time-critical.

The proportion of medicines reconciliation within 24-h of admission was analysed across the hospital between November 2020 and May 2021. During implementation, patients admitted Friday-Sunday were more likely to receive medicines reconciliation within 24-h (RR 1.41 (95% CI 1.34–1.47), *p* < 0.001). Rostered services were delivered sustainably in terms of shift-fill rate and medicines reconciliation outcome.

**Conclusion:**

Seven-day clinical pharmacy services benefit patient outcome through early medicines reconciliation and intervention. Investment to permanently embed the service was sustained.

## Facilitators of best practice

- A seismic surge in COVID-19 admissions gave urgent need for change in clinical services. This prompted re-deployment of pharmacists from clinical support roles to patient-facing clinical roles.
- Development of a service specification, alongside staff induction and training, defined the priorities for a novel weekend clinical pharmacy service. Weekly training continued throughout implementation to support clinical pharmacists (CP) working in new clinical specialities.
- Pre-existing digital infrastructure including electronic patient records, electronic prescribing and medicines administration, linkage to community care records, clinical pharmacy prioritisation support and dispensary automation supported the implementation of a seven-day clinical pharmacy service.

## Barriers to best practice

- Due to the implementation climate, communication with stakeholders was challenging. This was overcome through induction and communication with the multidisciplinary team (MDT) using several methods. Full consultation was essential to most effectively plan (and adapt) the subsequently supported permanent seven-day clinical pharmacy service.
- It was impossible to develop the service incrementally, build CP confidence and MDT knowledge. This was overcome by ensuring CPs supervision and access to consultant CPs, training materials and positive feedback to build confidence.
- Where staffing levels were lower (e.g. pharmacy technicians), staff contributed to the seven-day clinical pharmacy service on a voluntary overtime rather than via rostering core-hours. Voluntary overtime services were challenging to sustain. For a sustainable service, rostering core-hours was essential.

## Background

Patients admitted to acute hospitals at the weekend in England have increased mortality compared to those admitted on weekdays [1]. Several studies have reported this phenomenon known as ‘the weekend effect’ [2–4]. Although well documented, little is described regarding influencing factors.

Clinical pharmacists (CP) are essential for ensuring medicines are optimised and free from unintentional error [5–8]. This is well documented in older populations with multiple comorbidities where polypharmacy adds complexity, negatively impacts rehabilitation, patient outcome and causes re-admissions [9–12]. Medication optimisation (MO) not only supports patient safety but facilitates discharges and flow through healthcare [7]. Improved patient flow facilitates hospital efficiency; better serving local and specialist populations [13, 14]. CPs facilitate this by reconciling discharge medications on weekdays, and performing medicines reconciliation (MR) as a dynamic process [8, 15–18].

The Seven Days a Week Forum, established in 2013 in response to increasing evidence, reported significant variations in outcomes for emergency weekend admissions [19]. In 2016, NHS England described the need for transformative seven-day clinical pharmacy services, shifting away from the historical focus of dispensary services [20].

The first edition of the General Provision of Intensive Care Services (GPICS 2015), a publication from professions and organisations within the intensive care community, described ‘extension to a seven-day clinical pharmacy service’ as an unmet need for UK intensive care [21]. In 2016, PROTECTED ICU UK was undertaken in 21 critical care units in UK acute hospitals [22].Of the 21, 2 delivered a seven-day clinical pharmacy service. Clinical activity at these sites described an increase in CP intervention rate from 1 in 5 on weekdays to 1 in 3 at weekends [23]. In 2022, the UK Healthcare Safety Investigation Branch (HSIB) published an independent investigation into weight-based medication errors in paediatrics, following a fatal ten-fold dosing error. Authors recommended improvement in seven-day clinical pharmacy services [24].

In the UK, Royal Pharmaceutical Society (RPS) guidance recommends targeting services to ‘more complex patients during admission and discharge, thereby ensuring smooth transfer through care settings’ [7]. MR in acute hospitals should be complete within 24-h of admission, regardless of day, to ensure early action on discrepancies, and continuity of medication supply [25]. Technology should be used to support prioritisation of high risk or unstable patients [20].

Best practice is embedding medicines optimisation (MO) into routine care seven-days a week. In 2021, annual benchmarking of acute UK hospital pharmacy services indicated significant variation remained in seven-day clinical pharmacy provision [26]. Most hospitals provided a weekend clinical pharmacy service in acute assessment units (AAUs). Services beyond this, to critical care and high dependency units, were highly variable or absent [26].

There remains a paucity of published evidence describing implementation, clinical impact or sustainability of a seven-day clinical pharmacy service [27].

### Aim

The aim of this paper is to describe the development, implementation and evaluation of a seven-day clinical pharmacy service in a tertiary-referral London teaching hospital. We report effectiveness, feasibility and sustainability.

## Development

A seven-day clinical pharmacy service was developed in response to demand from senior leaders during the 2nd COVID-19 pandemic surge. Clinical support (e.g. education and training and formulary) pharmacists were temporarily redeployed as patient-facing CPs. All pharmacists were familiar with working as CPs, through regular clinical commitments. CPs were rostered to deliver a seven-day clinical pharmacy service, to support the multidisciplinary team (MDT) and strengthen MO in critical care, acute and general medical patients (including COVID-19), in accordance with national advice on acute sector workforce models during COVID-19 [28].

Development of the seven-day clinical pharmacy service is described with reference to the Consolidated Framework for Implementation Research (CFIR), a ‘meta-theoretical’ framework consisting of common constructs from published implementation theories. The framework describes four activities of implementation process: planning; engaging; executing; reflecting and evaluating [29].

## Planning

Planning and implementation was rapid, occurring over 2-weeks due to clinical need as a consequence of pandemic surge [30].

A number of existing infrastructures at King’s College Hospital (KCH) NHS Foundation Trust supported development of a seven-day clinical pharmacy service. These included: Electronic patient records (EPR), electronic prescribing and medicines administration (EPMA); patients’ community care records available via EPR and automation of dispensaries. An EPR report (‘WardView’) was available to support CP prioritisation. The report identified those patients who: had not received admission MR; had unresolved MR issues; were prescribed duplicate medicines; had omitted doses of medicines in previous 24-h; were prescribed higher risk medicines or had renal impairment.

The pharmacy workforce constituted consultant and expert CPs, medicines management pharmacy technicians (MMPTs) and pharmacy assistants. Approximately one-third of CPs were independent prescribers, who prescribed autonomously for conditions within their competence. Earlier in the COVID-19 pandemic, several experienced CPs were trained and re-deployed to a seven-day clinical pharmacy service in critical care and provided implementation feedback [31].

Planning was undertaken by clinical leads and consultant CPs, in consultation with dispensary leads. Activity data informed plans. Minimum staffing requirements for dispensaries were maintained.

Pre–intervention, weekend services were provided through overtime with limited patient-facing CP activities. During intervention, CPs at Denmark Hill site (DH) were rostered to a seven-day clinical pharmacy service as part of core hours. Lower staffing proportion and higher vacancy rate prevented rostering of CPs (excepting critical care) at Princess Royal University Hospital (PRUH) and MMPTs at both sites, due to negative impact on weekdays. These staff contributed on a voluntary overtime basis, additional to usual weekend commitments. Rosters were developed assuring CPs deployed in critical care worked alongside experienced CPs facilitating supervision and escalation [28].

Clinical leads developed a weekend service specification identifying priority groups for near-patient MO, using definitions from national guidance [7, 28]. This prioritised: critical care patients; patients deemed higher risk or unstable; patients referred by the MDT/weekday CP; and patients newly admitted or discharge-ready, including discharge medication counselling.

Due to pandemic implementation, full staff consultation was deemed unnecessary. Clinical leads delivered two induction sessions by secure video chat, attended by all CPs. Service specification and handover process were outlined, alongside matters important to stakeholders including roster pattern and pay. Specialist CPs developed weekly training sessions and aide-memoires to support CPs working outside usual clinical specialties.

Due to pandemic pressures, it was not feasible to implement the change incrementally, reducing opportunity to build CP confidence in novel clinical settings. Reassurance was provided at induction and plans made for dissemination of positive feedback.

## Implementation

### Engaging

The Chief Pharmacist acted as expert opinion leader, holding Deputy Chief Pharmacist, Clinical Services accountable as internal implementation leader. Associate Chief Pharmacists, Clinical Services co-led the project. A number of consultant and specialist CPs developed as change champions, supporting the intervention. Communication with MDT stakeholders was challenging due to rapid implementation and overwhelming burden of the COVID-19 pandemic.

### Execution

Implementation took place for eleven weeks, from 3rd January–14th March 2021, in adult critical care, AAUs and selected general adult wards (including COVID-19 wards) over KCH, covering approximately 50% of inpatients. CPs not delivering the seven-day clinical pharmacy service fulfilled usual pharmacy weekend services (Table 1).

**Table 1:**
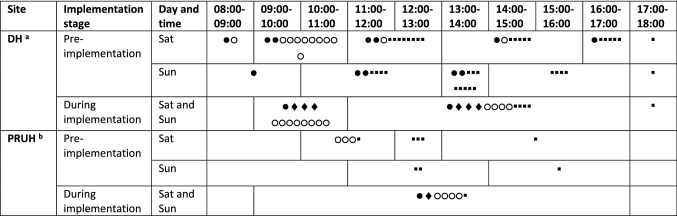
Distribution and working hours of pharmacists contributing to weekend pharmacy services before and during implementation

^a^DH–Denmark Hill site

^b^PRUH–Princess Royal University Hospital Site

●Ward-based acute admissions pharmacist, including post-take ward round and emergency care (post-take ward round suspended during implementation)

♦Ward-based critical care pharmacist

○Ward-based general adult pharmacist

▪Dispensary-based pharmacist

## Evaluation

A prospective observational study was conducted across areas receiving the seven-day clinical pharmacy service over consecutive weekends in February 2021. Workload prevented collection over the entire 11 weeks. The study was presented to Pharmacy Research and Audit Group and deemed a service evaluation without need for ethics approval.

### CP workforce, demographic and activity data

CP workforce and activity data were collected over the study period for prioritised areas. Data were collected by individual CPs using a validated CP activity recording form used quarterly over KCH [32]. Activity data included total number of CP patient reviews (referred or proactive), number of patient MRs and number of medicines that required amendment, medicines supply activities, CP independent prescribing, discharge activities and patient counselling.

All CP activity data were collated in the week immediately after included weekends. Data were inputted into a piloted case report form in Microsoft Excel (2016) by two CPs. Data queries were immediately resolved through direct contact with completing CPs. Data were analysed by the study team. Bed occupancy data for each clinical area were extracted from online analytical processing (OLAP) provided by KCH Business Intelligence Unit. In one critical care unit, OLAP data were not available and CP-reported bed occupancy accepted.

### Clinical contributions

Clinical contributions were self-reported by CPs delivering the seven-day clinical pharmacy service using a validated contributions form [33]. Patient identifiable data were anonymised. CP leaders received training from the principal investigator on form completion, who in turn trained their CP teams.

Clinical contributions were independently reviewed, categorised and severity graded by three pharmacists (Specialist Pharmacist (AW), Clinical Academic Research Lead (CM), Consultant Pharmacist (SJ)), using a methodology previously reported in PROTECTED ICU UK [22, 23].

Each contribution was categorised as error, optimisation or consult. A medication error was defined as an error in the process of prescribing, dispensing, preparing, administering, monitoring or providing medicine advice regardless of whether harm has occurred. Optimisation was defined as a proactive contribution that sought to enhance patient care. A consult was defined as a reactive intervention in response to MDT request [22, 23].

Each clinical contribution was independently assigned a clinical impact, of low, moderate, high or life threatening by AW and CM, applying the same methodology as PROTECTED-ICU UK [22, 23]. Where impacts matched, they were accepted; in disagreement, the third arbiter (SJ) assessed and score matching SJ’s assessment selected.

To assess the timeliness of CP contributions; a novel, categorical, time dependence scale was proposed by CM and Deputy Chief Pharmacist (CC). Time-dependency was defined as ‘the clinical benefit to patient or medicines safety risk of delivering the contribution on day of execution (e.g., Saturday) as opposed to 48-h hence (Monday)’. Guidance for this scale was given by evidenced based example (e.g. early introduction of beta-blocker post myocardial infarction or exposure to penicillin based antimicrobial in a patient with previous anaphylaxis) [34, 35]. Time-dependence was independently assessed by AW, CM and third arbiter, SJ, who engaged in disagreement.

### Medicines reconciliation (MR)

Data were collected from Allscripts Sunrise EPMA from 1st November 2020 to 31st May 2021. This provided a 2-month control period before implementation and a 2.5-month period after cessation. When MR was undertaken in wards (excluding critical care), CPs placed an EPMA MR order. A structured query language (SQL) report was built to report MR and admission date and time. All patients with an MR recorded on Allscripts Sunrise were captured, data were anonymised. MR and admission date and time was compared for each patient and percentage MR completed within 24-h calculated in intervention and control periods. It was not feasible to stratify data according to ward of admission, thus data for all admitted inpatients across DH and PRUH were compared.

### Analysis

Descriptive statistics was undertaken and analysed in IBM SPSS Statistics for Windows, premium version 28 and Microsoft Excel 2016. Categorical data were analysed using chi-square, with *p*-value < 0.05 considered significant.

On weekend 1, 8 CPs provided clinical pharmacy services across 29 wards and reviewed 369 patients. On weekend 2, 10 CPs provided clinical pharmacy services across 30 wards and reviewed 233 patients. Data were missing for 2 (3.3%) wards, both on weekend 2. No MMPTs contributed during evaluation (Table 2).

**Table 2:** Clinical pharmacist activities during data collection weekends

	Critical care units	Acute assessment units	General adult wards	Total
*Demographics*				
Occupied beds (n)	386	90	1108	1584
Patients who received CP review (n)	251	96	255	602
CP contributing to weekend clinical pharmacy service (n)	7	2	9	18
*Total activities interventions*				
Total CP activities (n)	**404**	**262**	**831**	**1497**
Interventions recorded (n)	**164**	**64**	**118**	**346**
Intervention rate per patient reviewed	0.65	0.67	0.45	0.57
Medical team acceptance of interventions (n (%))				
Accepted	149 (90.9)	37 (57.8)	90 (76.3)	276 (79.8)
Rejected	13 (7.9)	0 (0)	4 (3.4)	17 (4.9)
Undetermined acceptance	2 (1.2)	27 (42.2)	24 (20.3)	53 (15.3)
Categorisation of interventions (n (%))				
Medication errors	46 (28.0)	37 (57.8)	49 (41.5)	132 (38.2)
Optimisations	92 (56.1)	21 (32.8)	53 (44.9)	166 (48.0)
Consults	21 (12.8)	5 (7.8)	10 (8.5)	36 (10.4)
Consensus not reached	5 (3.0)	1 (1.6)	6 (5.1)	12 (3.5)
Grading of interventions (n (%))				
Life threatening	0 (0)	0 (0)	0 (0)	0 (0)
High	30 (18.3)	9 (14.1)	18 (15.3)	57 (16.5)
Moderate	116 (70.7)	42 (65.6)	81 (68.6)	239 (69.1)
Low	12 (7.3)	10 (15.6)	17 (14.4)	39 (11.3)
Consensus not reached	6 (3.7)	3 (4.7)	2 (1.7)	11 (3.2)
Interventions classified as time dependent (n (%))	101 (61.2)	29 (46)	66 (56.4)	196 (56.7)
*Medicines reconciliation*				
Admission MRs completed (n)	**28**	**48**	**101**	**177**
Patients where a change to the prescription was advised as a result of MR (n (%))	15 (53.6)	22 (45.8)	83 (82.2)	120 (67.8)
*Inpatient activities*				
Patients own drugs (PODs) assessed for re-use (n)	**2**	**26**	**73**	**101**
Inpatient medication orders (n)	**81**	**59**	**323**	**463**
Stock orders (n)	**0**	**28**	**78**	**106**
Patients counselled (n)	**0**	**8**	**15**	**23**
Inpatient items prescribed/de-prescribed by CP (n)	**129**	**15**	**69**	**213**
*Discharges*				
Discharges prepared (n)	**N/A**	**14**	**54**	**68**
Discharges where CP-led prescription/ transcription of medicine list (n %))	N/A	13 (92.9)	40 (74.1)	53 (77.9)

Items in bold add up to the total CP activities

*CP* Clinical pharmacist, *MR* medicines reconciliation

CPs conducted 251 CP reviews in critical care and 351 reviews in AAU or general adult wards. There were 172 new admissions during data collection (Table 2).

Admission MR was conducted for 177 patients. CPs recommended a change to prescription for 120/177 (67.8%) patients, for 227 prescribed medicines and assessed 101 packs of patient’s own medicines for re-use. MR data was incomplete for 2/102 data collection forms (Table 2).

CPs made 569 medication orders and prepared 68 discharge prescriptions. Of the 68, CPs led prescription/transcription of discharge medicine lists in 53 patients (77.9%), and clinically screened discharge prescriptions for 15 patients (22.1%); 23/68 patients (33.8%) received medicines counselling. CP independent prescribers prescribed or de-prescribed 213 inpatient medicines (Table 1).

The intervention rate per CP reviewed patient was 0.57; the rate was highest in AAUs and critical care (0.67 and 0.65 respectively). Of 346 CP interventions, 166 (48.0%) were optimisations, 132 (38.2%) errors, and 36 (10.4%) consults. Consensus was not reached on categorisation for 12 (3.5%) interventions (Table 2).

**Fig. 1:**
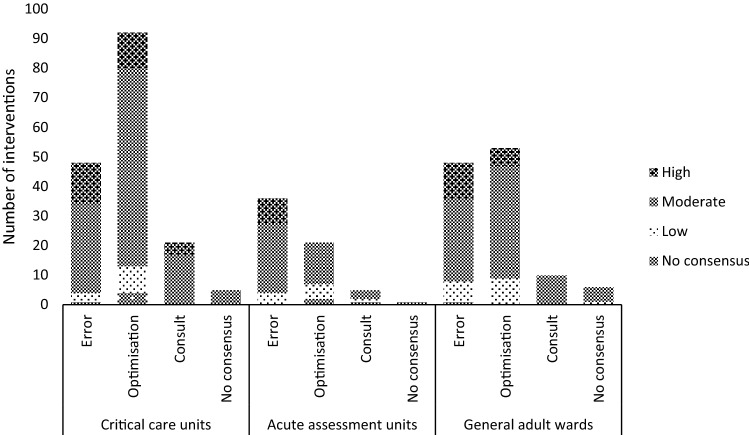
Number, type and impact of clinical pharmacist interventions by clinical area. Figure 1 is a bar chart illustrating the number of interventions broken down by error, optimisation consult and no consensus in critical care units, acute assessment units and general adult wards

Investigators classified 57 (16.5%) interventions as high impact, 239 (69.1%) moderate and 39 (11.3%) low. Consensus was not reached for 11 (3.2%) interventions (Fig. 1). Example interventions are given (Table 3).

**Table 3:** Example interventions, categorised by impact score, type and time-dependency

Example intervention	Impact score	Type	Time- dependent (Y/N)
Enteral ciprofloxacin prescribed twice daily for patient in critical care with continuous enteral feed. No allowance for required break in feed 2 h before and ciprofloxacin. Ciprofloxacin switched to intravenous to enable good absorption whilst continuing adequate feed	High	Optimisation	Y
Patient with advanced Parkinson’s disease (PD) usually managed with co-careldopa intestinal gel (Duodopa®) pump. Pump not working and patient not receiving PD medication on admission. Plan for alternative oral medication made and same prescribed	High	Error	Y
Patient enrolled in Tactic-R clinical trial (Reslizumab), consultant asked for information on the trial drug and if it should be continued. Information provided and confirmed one dose had been received already, and no further doses were indicated	High	Consult	Y
Omeprazole prescribed for gastroprotection during dexamethasone therapy. Dexamethasone course was complete and omeprazole stopped	Moderate	Optimisation	N
Patient co-prescribed amlodipine and simvastatin at dose > 20 mg, which is contrary to recommendations. Advised a switch to atorvastatin	Moderate	Error	N
Request for advice on switching from enoxaparin treatment dose to heparin infusion. Reviewed patient and advised omission of IV heparin bolus and to start heparin infusion at standard starting rate in guideline	Moderate	Consult	Y
Patient prescribed ibuprofen gel. Not on formulary or available. Formulary alternative (ketoprofen) recommended and supply made	Low	Optimisation	N
Calcium and vitamin D chewable tablets for bone protection not prescribed on admission. Same prescribed	Low	Error	N

**Fig. 2:**
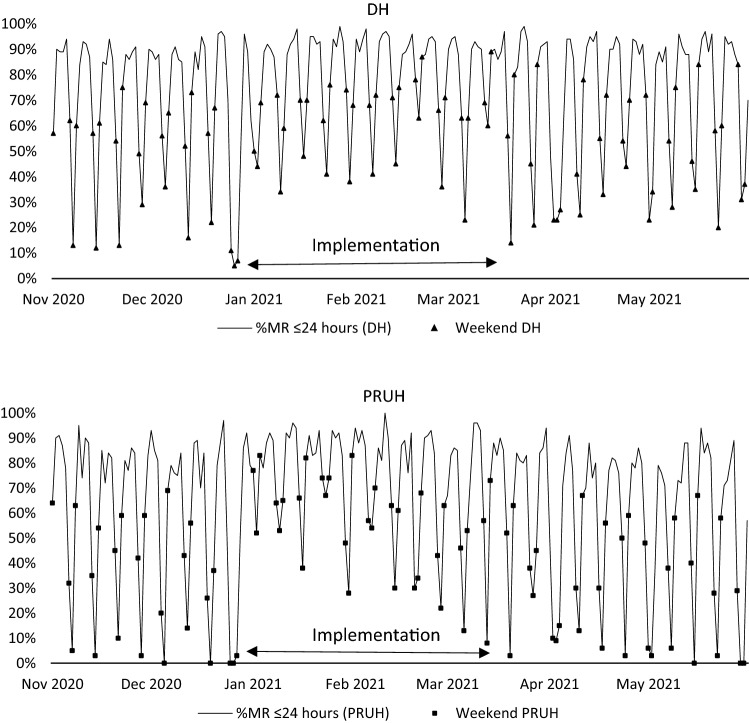
Time series of medicines reconciliation (MR) completion rates within 24-h of admission at Denmark Hill (DH) and Princess Royal University Hospital (PRUH). Figure 2 is a time series illustrating the percentage of patients receiving admission MR within 24-h on a daily basis between November 2020 and May 2021. Data points for days affected by weekend services (Friday-Sunday) are indicated

Four in five interventions (276/346, 79.8%) were accepted and 1 in 20 not accepted. In the remaining 53 (15.3%), acceptance was undetermined because of missing data (Table 1). Non-accepted interventions were optimisations for re-review at a later date, or were deemed appropriately rejected based on additional information held by the medical team. Investigators classified 196/346 (56.7%) of interventions as time-dependent. The time-dependent proportion was highest in critical care (101/164, 61.2%). Throughout implementation, patients admitted Friday–Sunday were more likely to receive MR within 24-h compared to control (RR 1.41 (95% CI 1.34–1.47), *p* < 0.001). The RR was highest in Saturday admissions, compared to control (RR 2.46 (95% CI 2.15–2.81%), *p* < 0.001) (Table 4). Reduction in variation of 24-h MR completion was sustained throughout DH implementation, but not at PRUH (Fig. 2).

**Table 4:** Comparison of medicines reconciliation completion rates within 24-h of admission during and the seven-day clinical pharmacy service period and control periods

Day of admission	During seven day clinical pharmacy service period		During control periods			
	Number of patients included	Number (%) patients receiving MR within 24 h	Number of patients included	Number of patients receiving MR within 24 h	RR of receiving MR within 24 h (95% CI)	*P* value^a^
Friday	972	612 (63)	1780	826 (46)	1.36 (1.26–1.46)	*P* < 0.001
Saturday	965	392 (41)	1537	254 (17)	2.46 (2.15–2.81)	< 0.001
Sunday	948	678 (72)	1786	1034 (58)	1.24 (1.16–1.32)	< 0.01
All weekend (Fri-Sun)	2885	1682 (58)	5103	2114 (41)	1.41 (1.34–1.47)	< 0.001
All weekdays (Mon-Thurs)	4816	4394 (91)	9883	8528 (86)	1.06 (1.03–1.08)	0.03
All days (Mon-Sun)	7381	5774 (78)	14,592	10,269 (70)	1.11 (1.09–1.14)	< 0.001

^a^The chi-square test was applied to assess significance

DH CP rostered services were fully-staffed throughout implementation. In PRUH CP volunteer rotas, shifts were consistently filled in weeks 1–4, but the fill-rate decreased to < 50% from week 5 onwards. Volunteer MMPT shifts were inconsistently filled throughout implementation.

## Discussion

A seven-day clinical pharmacy service was successfully developed, implemented and evaluated. The service was effective, with one intervention made in every second patient clinically reviewed. Previous systematic reviews and meta-analyses have demonstrated the impact of CP intervention on clinical outcome in terms of MR, preventing adverse drug events and prescribing errors in specific clinical contexts [36–42]. Clinical acceptability was demonstrated with an acceptance rate of 79.8%, comparable to published data (range 60–90%), despite 15.3% unrecorded intervention acceptance [23, 43–47]. Proportion of admission MR within 24-h increased, with greatest impact for Saturday admissions, reflecting significant changes made to the Saturday afternoon and Sunday services. Implementation provided capacity for CP-led preparation of weekend discharge medicines lists, which improves communication of discharge MR information to primary care [48].

This is the first study we are aware of to give direct evidence in terms of patient benefit for a seven-day clinical pharmacy service. We believe our findings provide proof of concept of an unmet need for a sustained seven-day clinical pharmacy service more broadly over the acute healthcare setting prioritised in critical care, acute and general medical settings; in addition to AAUs. Based on these findings, we developed and implemented a full seven-day clinical pharmacy service across KCH, demonstrating acceptability and sustainability.

Although we were unable to incrementally implement our service, it gave opportunity to rapidly build experience and assess the seven-day model, reflect on feasibility and opportunities for optimisation. We reflected on key facilitators and barriers influencing implementation and performance by considering all CFIR domains [29]. The implementation climate was considered a key facilitator. Rapid surge in COVID-19 admissions provided significant tension for change nationally and locally from senior stakeholders. This prompted re-deployment of clinical support CPs, providing additional resource to seven-day CP teams. Pharmacy leaders were fully engaged, CP change-champions supported and drove implementation of the seven-day clinical pharmacy service. Internal and external stakeholders shared the perception of the importance of development of seven-day clinical pharmacy services.

Despite rapid development, there was a high degree of readiness for implementation through existing infrastructures. At induction, a minority of CPs described low self-efficacy in delivery of the service specification; this is recognised as a key determinant in implementation literature [49, 50]. This was overcome through access to expert and consultant CPs, regular positive feedback through dissemination of key performance indicators and recognition from KCH executive team. Expert and consultant CPs delivering bite-size training and learning resources were crucial. The weekend service was facilitated by a service specification which defined priority patient groups. The ‘WardView’ report supported CP’s clinical prioritisation. This is a locally developed tool without formal evaluation. There is a paucity of literature in CP prioritisation tools and in our opinion, stronger data would support implementation of seven-day clinical pharmacy services [51]. We considered a mature EPR/EPMA system with linkage to community care records to be beneficial for effective implementation of out of hours MR [52, 53].

The seven-day clinical pharmacy service was adapted over DH and PRUH to roster teams as part of core hours or a voluntary overtime basis. Shift-fill rate and MR data indicated that services delivered by rostered staff in core hours were sustainable. Services delivered by voluntary overtime waned as organisational pressure decreased and volunteers fatigued.

### Limitations and recommendations for future research

Services were not developed or evaluated in our full range of clinical specialties, including neurosciences, hepatology, paediatrics or maternity for this intervention. The impact of implementation on dispensary services was not measured, although anecdotally dispensaries ran smoothly. It was not feasible to assess direct patient outcomes in-terms of length of stay, mortality or re-admission due to rapid implementation and the confounding effect of the COVID-19 pandemic.

Cost was not assessed because of clinical context. Robust economic evaluation was essential for planning and scale-up to permanent service.

The seven-day clinical pharmacy service was assessed over just 2 weekends and during the COVID-19 pandemic which limited generalisability of findings. The novel time-dependence scale developed was not validated. Additionally, it was challenging to collect and analyse MR data with differing EPMA systems, although this did not affect service delivery.

CFIR was adopted retrospectively, and thus we did not fully explore all CFIR constructs. Further quantitative and qualitative evaluation over a range of specialities, supported by prospective use of implementation frameworks to fully explore underpinning implementation theory should be our next steps in providing more comprehensive evaluation of seven-day clinical pharmacy services.

## Conclusion

A seven-day clinical pharmacy service was successfully implemented and was effective in impacting clinical outcome through CP intervention and rapid MR in acutely unwell patients in a tertiary-referral London teaching hospital. Key facilitators and barriers of implementation were identified using implementation frameworks. These findings support CPs in acute sectors implementing clinical pharmacy services to our patients seven-days a week.
